# Characterization of Maize Hybrids (*Zea mays* L.) for Detecting Salt Tolerance Based on Morpho-Physiological Characteristics, Ion Accumulation and Genetic Variability at Early Vegetative Stage

**DOI:** 10.3390/plants10112549

**Published:** 2021-11-22

**Authors:** Md Al Samsul Huqe, Md Sabibul Haque, Ashaduzzaman Sagar, Md Nesar Uddin, Md Alamgir Hossain, AKM Zakir Hossain, Md Mustafizur Rahman, Xiukang Wang, Ibrahim Al-Ashkar, Akihiro Ueda, Ayman EL Sabagh

**Affiliations:** 1 Department of Crop Botany, Bangladesh Agricultural University, Mymensingh 2202, Bangladesh; sagor325@gmail.com (M.A.S.H.); sagar@bau.edu.bd (A.S.); nesar.uddin@bau.edu.bd (M.N.U.); alamgircbot@bau.edu.bd (M.A.H.); zakir@bau.edu.bd (A.Z.H.); chairman.pcrf@gmail.com (M.M.R.); 2 Department of Biology, College of Life Sciences, Yan’an University, Yan’an 716000, China; 3 Department of Plant Production, College of Food and Agriculture, King Saud University, Riyadh 11451, Saudi Arabia; ialashkar@ksu.edu.sa; 4 Graduate School of Integrated Science for Life, Hiroshima University, 1-4-4 Kagamiyama, Higashi-Hiroshima 739-8528, Japan; akiueda@hiroshima-u.ac.jp; 5 Agronomy Department, Faculty of Agriculture, Kafrelsheikh University, Kafrelsheikh 33516, Egypt

**Keywords:** salt tolerance index, principal component analysis, plant biomass, ion accumulation, maize hybrid, heritability

## Abstract

Increasing soil salinity due to global warming severely restricts crop growth and yield. To select and recommend salt-tolerant cultivars, extensive genotypic screening and examination of plants’ morpho-physiological responses to salt stress are required. In this study, 18 prescreened maize hybrid cultivars were examined at the early growth stage under a hydroponic system using multivariate analysis to demonstrate the genotypic and phenotypic variations of the selected cultivars under salt stress. The seedlings of all maize cultivars were evaluated with two salt levels: control (without NaCl) and salt stress (12 dS m^−1^ simulated with NaCl) for 28 d. A total of 18 morpho-physiological and ion accumulation traits were dissected using multivariate analysis, and salt tolerance index (STI) values of the examined traits were evaluated for grouping of cultivars into salt-tolerant and -sensitive groups. Salt stress significantly declined all measured traits except root–shoot ratio (RSR), while the cultivars responded differently. The cultivars were grouped into three clusters and the cultivars in Cluster-1 such as Prabhat, UniGreen NK41, Bisco 51, UniGreen UB100, Bharati 981 and Star Beej 7Star exhibited salt tolerance to a greater extent, accounting for higher STI in comparison to other cultivars grouped in Cluster-2 and Cluster-3. The high heritability (h^2^_bs_, >60%) and genetic advance (GAM, >20%) were recorded in 13 measured traits, indicating considerable genetic variations present in these traits. Therefore, using multivariate analysis based on the measured traits, six hybrid maize cultivars were selected as salt-tolerant and some traits such as Total Fresh Weight (TFW), Total Dry Weight (TDW), Total Na^+^, Total K^+^ contents and K^+^–Na^+^ Ratio could be effectively used for the selection criteria evaluating salt-tolerant maize genotypes at the early seedling stage.

## 1. Introduction

Salinity above its tolerable limits is one of the mainly detrimental ecological conditions, resulting in a significant reduction in plant development and productivity [1,2]. High salinity affects around 50% of the world’s irrigated land and one-fifth of cultivable land [3]. Both arid and semi-arid regions bear the burden of salinity’s detrimental effects due to decreased precipitation [4]. This process enhanced the concentration of salts both in the land and in plants. Generally, saline soil is referred to as one in which the electrical conductivity (EC) exceeds 4 dS m^−1^ at 25 °C in the root zone of the crop [5]. The yield of most crop plants is reduced at this EC, though some crops exhibit yield reduction even at lower EC than 4 dS m^−1^ [6]. Around 62% of Bangladesh’s coastal areas are affected to varying degrees by soil salinity [7] because of tidal surges during the rainy season, direct flooding by storm surges, and salt movement in the ground and surface water during the dry season [8]. Moreover, about 30% of the cultivable lands of coastal areas are affected by salinity in Bangladesh. The salt concentration in the soil of these areas increases gradually due to the use of shallow saline groundwater resources for irrigation. Moreover, the climate model predicts that the area of saline soils (>1 ppt, 16,720 sq km) in the southern region of Bangladesh will be increased by 14% (19,075 sq km) by 2050 due to sea-level rise and no upstream of freshwater inflow [9]. Thus, ensuring food security for the regularly increasing populations of Bangladesh is highly challenging. Generally, soil salinity affects plants in two ways; firstly, high NaCl accumulation outside the roots, which reduces the water potential of the soil solution leading to reduced water uptake by roots, resulting in physiological drought [2,10]. Secondly, a higher accumulation of Na^+^ and K^+^ in the plant tissues causes oxidative stress by producing reactive oxygen species (ROS) in the crop plants [11,12,13]. However, salt stress induces a variety of responses in plants, including alterations in morphological, physiological and biochemical characteristics [14,15]. For instance, the germination process of different crop plants has become hindered considerably due to ion toxicity under salinity stress [16,17] which further results in less water availability, stunted growth, accumulation of Na^+^ in tissues, less gaseous exchange and nutrient uptake, as well as failure in crop yield [2,18]. High Na^+^ accumulation in leaves causes stomatal closure and malfunctioning of the photosynthetic apparatus and electron transport chain, which leads to decreased photosynthesis and productivity [19,20]. Besides this, hyperosmotic levels of sodium and chloride ions in the root zone reduce the availability of potassium and calcium ions to the root, which leads to the accumulation of toxic Na^+^ in leaves [19,21], resulting in cell damage and the inhibition of enzyme and protein synthesis [22,23]. To cope with this adverse effect of salinity stress, plants accumulate different compatible osmolytes such as proline, glycine and betaine, etc. [24], which reduces the osmotic potential of cells and enables water absorption [25,26].

Maize (*Zea mays* L.) is an important C_4_ plant from the Poaceae family and is moderately sensitive to salt stress [27]. It ranks third among the most vital cereal crops that offer staple food to millions of people worldwide, providing half of all energy consumption [28]. Moreover, it is used as a major raw material in the textile, paper and feed industries [29,30]. It has been reported that soil salinity is one of the significant threats to maize production worldwide [31]. In general, maize shows decreased germination rate, stunted growth, reduced photosynthesis and less productivity under salinity stress [32,33]. However, maize production needs to be doubled, even under salinity stress, by 2050 to meet the ever-increasing demand of maize as a fundamental foodstuff [34]. The introduction of salt-tolerant maize genotypes and selection of existing suitable maize cultivars would be the better options to meet the challenge of increasing food demand. However, conventional breeding requires a long time to develop a suitable salt-tolerant crop. Thus, mass screening of existing maize genotypes is one of the possible ways to mitigate or adapt salt stress. A comprehensive understanding of the integration and trade-off between morphological, physiological and biochemical traits and their responses during the plant life cycle is important for inexpensive, fast and efficient detection of sensitive and tolerant genotypes [35,36]. Laboratory experiments for salt tolerance detection at germination or at the early seedling stage in a hydroponic growth system may be useful as it allows the precise control of salt concentration in the medium and to provide accurate data [37,38]. Field experiments lack accuracy in measuring root traits, require substantial phenotypic data for morphological measurements and need seasonal or yearly data repetition. Multivariate analysis is a useful tool for detecting the relationship between a wide range of variables and identifying genetic variations using multiple selection criteria. Multivariate analysis has been widely used to determine secondary characters and genotypic selection in many crops under salt stress [39,40,41,42,43]. As mentioned above, large parts of coastal areas in Bangladesh have suffered from soil salinity with an increasing trend and, thus, crop production is becoming challenging there. Maize production nowadays in Bangladesh is promising throughout the country and plenty of hybrid varieties are available in the market. Mass screening of these varieties for salt tolerance would be a possible way to select suitable varieties for cultivation in the coastal areas of Bangladesh. Therefore, this study was formulated to screen 18 prescreened, popular, high-yielding maize hybrids at the early seedling stage for the selection of suitable varieties to be cultivated in the saline coastal areas in Bangladesh. Moreover, diverse morpho-physiological and biochemical traits were dissected using multivariate analysis to identify useful traits to be used for further selection and improvement of salt-tolerant maize cultivars.

## 2. Materials and Methods

### 2.1. Plant Materials and Growth Conditions

**Figure 1:**
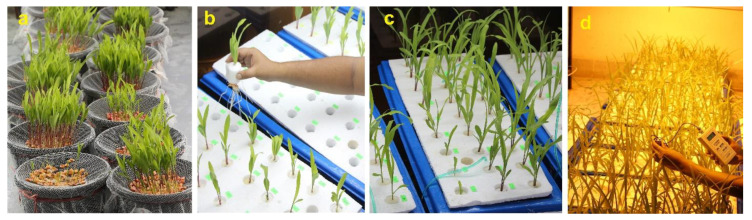
Photographs showing (**a**) germination of maize seeds, (**b**) placing of germinated seedlings in the hydroponic trays, (**c**) seedling growth in the hydroponic system and (**d**) light intensity measurements inside the growth chamber.

A set of 18 popular maize (*Zea mays* L.) hybrid cultivars were tested for salt tolerance in a hydroponic trial (Table 1). These 18 maize cultivars were selected from a prescreening experiment using 33 maize genotypes based on germination capability to salt stress [44]. The seeds of all cultivars were collected from different seed companies and suppliers in Bangladesh. The experiment was carried out in the growth chamber inside the Plant Physiology Laboratory, Department of Crop Botany, Bangladesh Agricultural University, Mymensingh, Bangladesh, in 2017. The collected seeds were sterilized with 5% sodium hypochlorite for 30 min and washed a few times with distilled water. After proper rinsing, the seeds were stored in a refrigerator and brought back to room temperature the day before sowing. The seeds were sprouted by placing them into a net and the net being kept with a gentle touch of water inside a bucket of filled water (Figure 1).

The hydroponic system was maintained in rectangular plastic tanks (32″ × 13″ × 9″; L × W × H). Perforated cork sheets were used as trays having 27 holes (3 × 9) per tray in each tank (Figure 1). Pregerminated five-day-old seedlings of all cultivars were placed in the holes of the tanks (32 L water in each tank) using a piece of Styrofoam to fix the young plants. After five days, the seedlings were allowed to grow on modified Hoagland’s nutrient solution (pH 5.5–6) with following composition: Ca(NO_3_)_2_·4H_2_O (2 mM), KH_2_PO_4_ (0.2 mM), K_2_SO_4_ (1 mM), CaCl_2_·2H_2_O (2 mM), MgSO_4_·7H_2_O (0.5 mM), Fe-EDTA (200 µM), H_3_BO_3_ (1 µM), CuSO_4_·5H_2_O (0.3 µM), MnSO_4_·6H_2_O (2 µM), (NH_4_)_6_Mo_7_O_24_ (0.01 µM), ZnSO_4_·7H_2_O (0.5 µM). Salinity was imposed after four days of adding nutrients in the tanks and kept for 14 days to observe salt tolerance. The seedlings of all cultivars were evaluated under two growing conditions: control (0 dS m^−1^) and salt stress (12 dS m^−1^). The salt level was obtained by dissolving laboratory-grade NaCl into the nutrient solution until the electrical conductivity (EC) reached 12 dS m^−1^ with the help of an EC meter, while the control medium only received the nutrient solution. The experiment was followed by a completely randomized design (CRD) with nine replications. Each tank was accommodated by three hills of nine cultivars (27 hills). Each growth condition consisted of six tanks (replicated thrice), and, thus, 12 tanks consisting of 324 plants (18 × 2 × 9) were used. A photoperiod of 16 h was maintained, providing artificial lighting using fluorescent lamps (40 W) at the initial stage. Later, high-pressure sodium lamps (HPS, 400 W) were used, and approximately 350 µmol m^−2^ s^−1^ of Photosynthetic Photon Flux Density (PPFD) was provided for the seedling growth. An optimum growing temperature (30 ± 2 °C day and 25 ± 1 °C night) was maintained during the experiment. The nutrient solution was replaced once a week and aerated continuously using individual air pump for each tank.

### 2.2. Morphological Measurements

The 28-day-old seedlings of all genotypes grown under control and salt conditions were harvested and the morphological data were recorded. Roots and shoots were separated, and root length (RL) and shoot length (SL) were measured (cm) using a one-meter ruler. Fresh weight of roots and shoots (RFW and SFW, respectively) were obtained using a digital balance (g). Then the roots and shoots were oven-dried at 70 °C for 72 h and the root dry weight (RDW) and shoot dry weight (SDW) in grams were determined. The total fresh (TFW) and dry weight (TDW) were calculated by the summation of RFW and SFW, and RDW and SDW, respectively. Root–Shoot ratio (RSR) was calculated as root dry weight over shoot dry weight. The morphological data were taken from three biological replicates of each treatment.

### 2.3. Physiological Measurements

The leaf greenness (relative chlorophyll content expressed in SPAD value) of the 28-day-old plants of each cultivar in all growth conditions was measured using a handheld portable chlorophyll meter (SPAD-502 Plus, Konica Minolta, Osaka, Japan). The reading was taken at the top, middle and bottom of the leaves and average values of those three readings were used as a single replicate value. The rate of photosynthesis (*A*) was measured in the first bottom true leaf of the plants in all growth conditions. Both SPAD and *A* measurements were recorded in three leaves (from three individual plants) of each treatment. The *A* measurements were performed by a portable photosynthetic system (LC*i*–SD photosynthetic system, ADC BioScientific Ltd., Hertfordshire, UK) at a PPFD of 300 µmol m^−2^ s^−1^ and ambient temperature in the growth room. The CO_2_ level was maintained as 400 ppm during *A* determination.

### 2.4. K^+^ and Na^+^ Analysis

The root and shoot dry samples of 21-day-old maize seedlings of all cultivars were collected to analyze the K^+^ and Na^+^ content. The K^+^ and Na^+^ elemental analysis was performed on acid digested material through micro-Kjeldahl digestion system with a slight modification [45]. Approximately 0.5 g dry materials were mixed with 5 mL 68% HNO_3_ in a digestion tube, mixed well and the tubes left overnight. Digestion of the samples was performed at 125 °C temperature for 4 h after boiling had started. After cooling, the digestion mixtures were taken into a 100 mL volumetric flask and the volume made up to 100 mL with distilled water. The mixtures were then filtered, and filtrates were stored into a screw cap dry bottle for analysis. Then 10 mL of filtrate was taken and the volume made up to 50 mL with distilled water in a volumetric flask and mixed properly. The concentrations of Na^+^ and K^+^ (mg g^−1^ DW) in three replicates of each treatment were measured by a flame photometer (Jenway-PFP7, Cole-Parmer, Staffordshire, UK).

### 2.5. Statistical Analysis

The open-source statistical software R [46] version 4.0.5 was used to compare the means between the treatments and among the cultivars. The salt tolerance index (STI) of each trait was calculated as stress/control × 100. Data analysis was conducted considering two-way (cultivar and salt stress) and one-way (cultivar with STI values) analysis of variance (ANOVA) with a significant level of *p* < 0.05. The multiple comparisons of treatment means and STI values among cultivars were performed by the Tukey HSD test of the R program. The standardized STI values were used to construct a two-way hierarchical clustering heatmap using the package *ComplexHeatmap* in R. The functions *ggpair* and *fviz_pca* of R were used to generate correlation–matrix scatter plot and principal component analysis (PCA) biplot. The genotypic (σ^2^_g_), genotype × salinity (σ^2^_gs_), residual (σ^2^_e_) and phenotypic (σ^2^_p_) variances were computed from the respective mean squares as described by [47,48,49].

(1) $$(\sigma^{2}{}_{g}=\frac{{\mathrm{MS}}_{g}{\;-\;\mathrm{MS}}_{\mathrm{gs}}}{\mathrm{rl}};{\;\sigma }^{2}{}_{\mathrm{gs}}=\frac{{\mathrm{MS}}_{\mathrm{gs}}{\;-\;\mathrm{MS}}_{e}}{r}\;;{\;\sigma }^{2}{}_{e}{=\mathrm{MS}}_{e};{\;\sigma }^{2}{}_{p}{=\sigma }^{2}{}_{g}+\frac{\sigma^{2}{}_{\mathrm{gs}}}{l}+\frac{\sigma^{2}{}_{e}}{\mathrm{rl}}$$

where MS_g_ = mean square of genotype, MS_gs_ = mean square due to genotype by salinity interactions, MS_e_ = error mean square, l = number of salt levels, r = number of replications.

The broad sense heritability (h^2^_bs_) was estimated using the method described in [50].

(2) $${\;h}^{2}{}_{\mathrm{bs}}=\frac{\sqrt{\sigma^{2}{}_{g}}}{\sqrt{\sigma^{2}{}_{p}}}\;\;\times \;100$$

The heritability was categorized as low (0–30%), moderate (30–60%) and high (>60%) following [51]. The genotypic coefficient of variability (GCV) and phenotypic coefficient of variability (PCV) were calculated according to the procedure outlined by [52].

(3) $$\mathrm{GCV}=\frac{\sqrt{\sigma^{2}{}_{g}}}{\bar{x}}\;\times \;100;\;\mathrm{PCV}=\frac{\sqrt{\sigma^{2}{}_{p}}}{\bar{x}}\;\times \;100$$

where $\bar{x}$ is the phenotypic grand mean for each trait.

The genetic advance (GA) and genetic advance as percent of the mean (GAM) were estimated following the formula suggested by [52,53].

(4) $$\mathrm{GA}(\%)={h^{2}}_{\mathrm{bs}}\times \sigma_{p}\times k;\;\mathrm{GAM}(\%)=\mathrm{GA}/\bar{x}\times 100$$

where σ_p_ = phenotypic standard deviation, k = selection differential at 5% selection intensity. The value of k is 2.06. GAM was classified and rated based on the scales given by [52] as low (<10%), moderate (10–20%) and high (>20%).

## 3. Results

### 3.1. Variability in Cultivars and Traits

**Figure 2:**
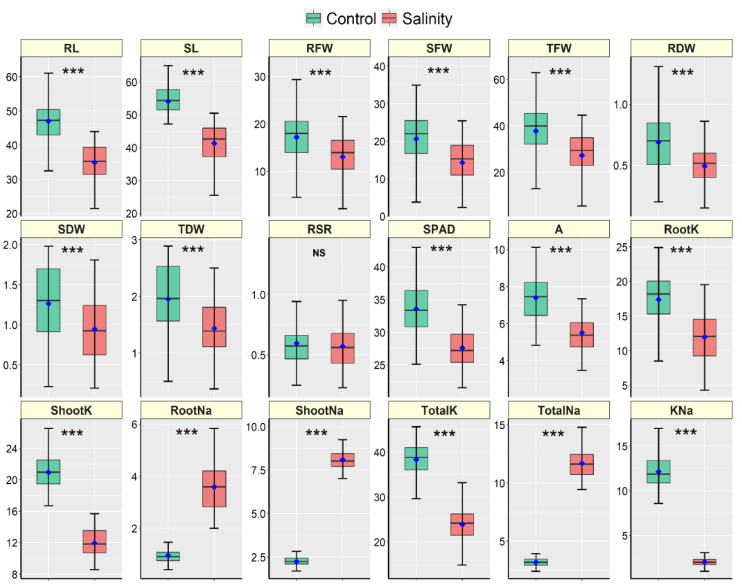
Boxplots illustrating the descriptive statistics of morpho-physiological and biochemical measured traits in 21-day-old seedlings of 18 hybrid maize cultivars grown in control and salt conditions under a hydroponic system. The blue points are treatment means, and the horizontal lines dividing the box represent the medians. The lower and upper box boundaries, as well as the lower and higher whiskers, reflect the Q1 (25th percentile), Q3 (75th percentile), minimum (Q1–1.5IQR) and maximum (Q1 + 1.5 IQR) values, respectively. IQR denotes Interquartile Range. *** and NS denote significant variation between treatments at 0.1% levels of probability and non-significant, respectively. Units of traits are as follows: RL (Root Length, cm), SL (Shoot Length, cm), RFW (Root Fresh Weight, g), SFW (Shoot Fresh weight, g), TFW (Total Fresh Weight, g), RDW (Root Dry Weight, g), SDW (Shoot Dry Weight, g), TDW (Total Dry Weight, g), RSR (Root–Shoot Ratio), SPAD (Leaf Greenness), A (Photosynthesis rate, µmol CO_2_ m^−2^ s^−1^), RootK (Root K^+^ concentration, mg g^−1^ DW), ShootK (Shoot K^+^ concentration, mg g^−1^ DW), TotalK (Total K^+^ concentration, mg g^−1^ DW), RootNa (Root Na^+^ concentration, mg g^−1^ DW), ShootNa (Shoot Na^+^ concentration, mg g^−1^ DW), TotalNa (Total Na^+^ concentration, mg g^−1^ DW), KNa (K^+^–Na^+^ Ratio).

The 18 cultivars responded differently in response to salt stress and considerable variations between the control and salt treatments were observed in almost all traits (Table S1; Figure S1 and Figure 2). The cultivars did not show significant variation in Shoot K^+^ concentration (ShootK) while the difference in root–shoot ratio (RSR) between the stress treatments was statistically similar (Table S1). The interaction effects (cultivar × stress) were significant in all traits except RSR and Shoot K^+^ concentration (Table S1). Figure 2 shows the descriptive statistics of all traits measured in 18 maize hybrid cultivars. All morpho-physiological traits except RSR were significantly declined due to salt stress in all cultivars (Figure 2). In the case of ion accumulation, root, shoot and total Na^+^ concentrations were increased due to salt stress. In contrast, the K^+^ concentrations in the root, shoot and whole plant were significantly declined under salt stress compared to the control in all maize cultivars (Figure 2).

### 3.2. Clustering of Cultivars and Traits Based on STI

**Figure 3:**
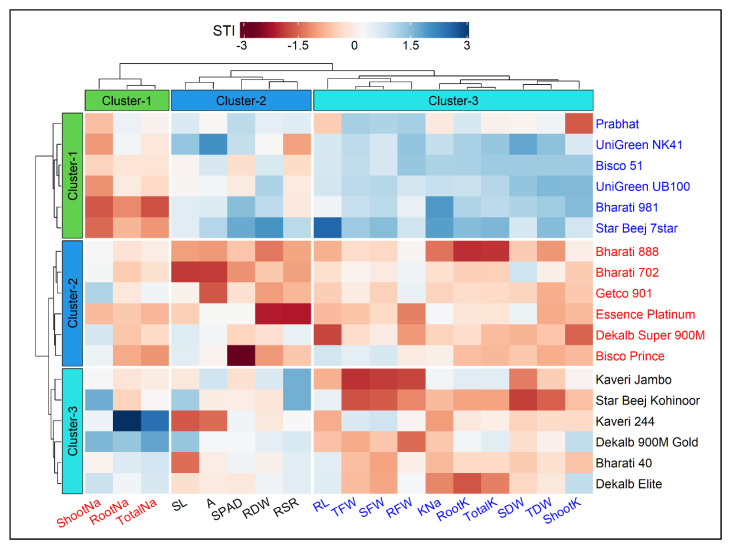
Hierarchical clustering and heatmap illustrating the associations among 18 maize cultivars and 18 different traits under salt condition. Each column represents a trait, whereas each row represents a cultivar. The different colors and intensities were adjusted based on cultivars–traits relationships. Colors are representative of a relative scale (−3 to +3) derived from data standardization of the STI (salt tolerance index) values. The darker red indicates lower values (salt−sensitive), while the darker blue indicates higher values (salt−tolerant). Both the cultivars and traits were grouped into three clusters each. RL (Root Length, cm), SL (Shoot Length, cm), RFW (Root Fresh Weight, g), SFW (Shoot Fresh weight, g), TFW (Total Fresh Weight, g), RDW (Root Dry Weight, g), SDW (Shoot Dry Weight, g), TDW (Total Dry Weight, g), RSR (Root–Shoot Ratio), SPAD (Leaf Greenness), A (Photosynthesis rate, µmol CO_2_ m^−2^ s^−1^), RootK (Root K^+^ concentration, mg g^−1^ DW), ShootK (Shoot K^+^ concentration, mg g^−1^ DW), TotalK (Total K^+^ concentration, mg g^−1^ DW), RootNa (Root Na^+^ concentration, mg g^−1^ DW), ShootNa (Shoot Na^+^ concentration, mg g^−1^ DW), TotalNa (Total Na^+^ concentration, mg g^−1^ DW), KNa (K^+^–Na^+^ Ratio).

The hierarchical clustering heatmap of cultivars and traits along with the dendrogram is presented in Figure 3. The cultivars and traits were clustered using the salt tolerance index (STI) values, and the optimum number of clusters was determined by the gap statistic method prior to clustering. Based on the variations that existed in the traits, the 18 maize cultivars were grouped into three row-clusters, and each cluster consisted of six closely associated cultivars (Figure 3). Similarly, the traits were grouped into three column-clusters, where Cluster-1, Cluster-2 and Cluster-3 comprised 3, 5 and 10 traits, respectively (Figure 3). The highly related traits such as Shoot Na^+^, Root Na^+^ and Total Na^+^ were assembled in Cluster-1; SL, *A*, SPAD, RDW and RSR in Cluster-2; RL, TFW, SFW, RFW, K^+^–Na^+^ ratio, Root K^+^, Total K^+^, SDW, TDW and Shoot K^+^ were segmented in Cluster-3 (Figure 3).

**Figure 4:**
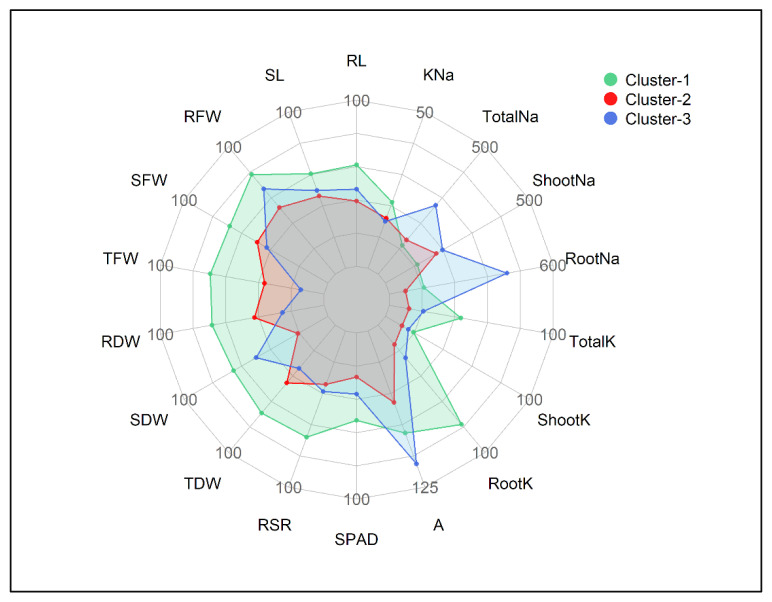
Radar plot showing salt tolerance index (STI) values of different traits in three clusters of 18 maize cultivars. The STI values are expressed as % of the control. The lowest to highest STI scales are 50 to 100 in all traits except K^+^–Na^+^ ratio (0–50), A (50–125), Shoot and Total Na^+^ (300–500) and Root Na^+^ (300–600), respectively. Units of traits are as follows: RL (Root Length, cm), SL (Shoot Length, cm), RFW (Root Fresh Weight, g), SFW (Shoot Fresh weight, g), TFW (Total Fresh Weight, g), RDW (Root Dry Weight, g), SDW (Shoot Dry Weight, g), TDW (Total Dry Weight, g), RSR (Root–Shoot Ratio), SPAD (Leaf Greenness), A (Photosynthesis rate, µmol CO_2_ m^−2^ s^−1^), RootK (Root K^+^ concentration, mg g^−1^ DW), ShootK (Shoot K^+^ concentration, mg g^−1^ DW), TotalK (Total K^+^ concentration, mg g^−1^ DW), RootNa (Root Na^+^ concentration, mg g^−1^ DW), ShootNa (Shoot Na^+^ concentration, mg g^−1^ DW), TotalNa (Total Na^+^ concentration, mg g^−1^ DW), KNa (K^+^–Na^+^ Ratio).

Based on the STI values, the cultivars in Cluster-1 exhibited greater salt tolerance followed by Cluster-3 and Cluster-2 (Figure 3 and Figure 4). Out of 18 traits, the lowest STI values were reflected by the traits viz. Shoot Na^+^, Root Na^+^ and Total Na^+^. In contrast, higher tolerance to salt stress was reflected by greater STI values in the other 15 traits. The STI values of RL, SL, RFW, SDW, RSR, SPAD, Root K^+^, Shoot K^+^ and Total K^+^ were higher in Cluster-1 followed by Cluster-3 and Cluster-2 (Figure 4). The STIs for TDW and RSR ranged from 67–85% and 67–84%, respectively, throughout the three clusters. The STI of *A* was recognized higher in Cluster-3 (114%) and lower in Cluster-2 (85%). Considering the STI of SFW, RFW, RDW, TDW and K^+^–Na^+^ ratio, the clusters were ranked as Cluster-1 > Cluster-2 > Cluster-3 (Figure 3 and Figure 4). The maximum STI in Total Na^+^ was observed in Cluster-3 (408%) followed by Cluster-2 (354%) and Cluster-1 (345%). The STI of K^+^–Na^+^ ratios were 21%, 16% and 15% in Cluster-1, Cluster-2 and Cluster-3, respectively (Figure 4). The heatmap clearly illustrates that the cultivars in Cluster-1, such as Prabhat, UniGreen NK41, Bisco 51, UniGreen UB100, Bharati 981 and Star Beej 7Star, shared higher STI values than those in Cluster-2 and Cluster-3. The lower STI from trait Cluster-1 indicates that these cultivars were salt-tolerant in comparison to the other cultivars that belonged to Cluster-2 and Cluster-3 (Figure 3).

### 3.3. Cluster Means of Traits under Control and Salt Treatments

The mean values of the measured traits of three clusters under control and salt conditions are presented in Table 2. All three clusters of 18 maize cultivars varied considerably. In general, salt stress significantly affected all traits in all clusters, but the cultivars under Cluster-1 performed better, showing greater salt values in comparison to other clusters in all traits except *A*, Root Na^+^, Shoot Na^+^, and Total Na^+^ (Table 2). Salt stress significantly increased the Root Na^+^, Shoot Na^+^ and Total Na^+^ contents compared to the control in all cultivars and their formed clusters. The net photosynthesis (*A*) in control plants ranged from 7.0 to 7.9 µmol CO_2_ m^−2^s^−1^, while *A* ranged from 5.2 to 6.0 µmol CO_2_ m^−2^s^−1^ in the salt–stressed plants in the three clusters (Table 2). The maximum K^+^–Na^+^ Ratio under the control condition was observed in Cluster-3 (12.5), which was statistically similar to Cluster-1 (12.4). In contrast, under salt stress, the K^+^–Na^+^ Ratio was calculated as 2.6, 1.8 and 1.9 in Cluster-1, Cluster-2 and Cluster-3, respectively (Table 2).

### 3.4. Principal Component Analysis (PCA)

**Figure 5:**
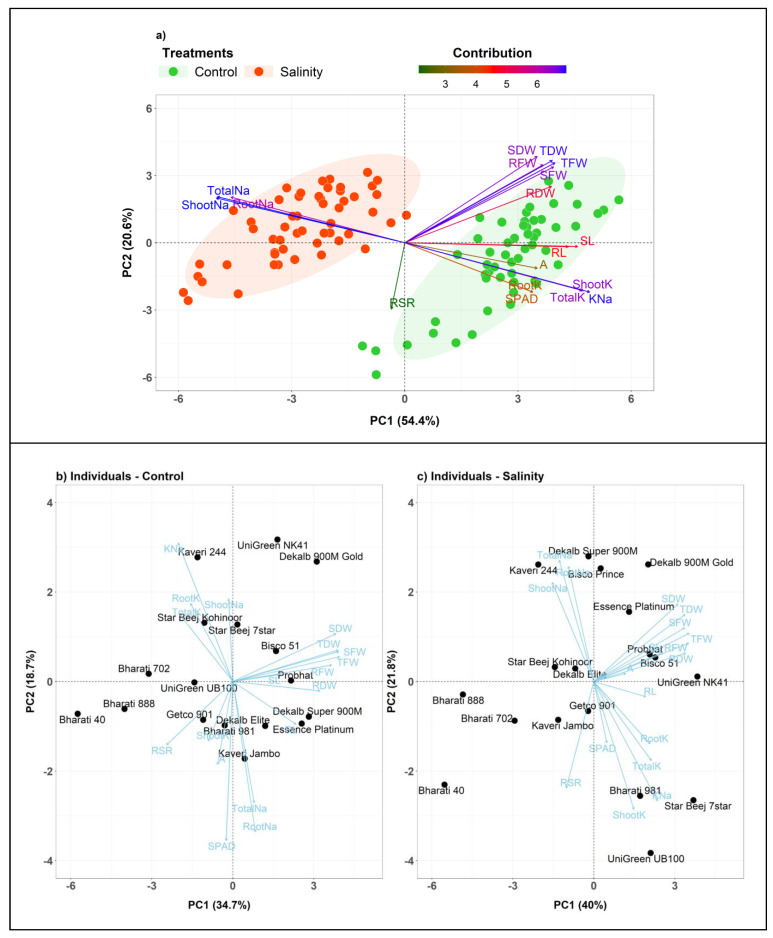
(**a**) Principal Component Analysis (PCA)−biplot of 18 maize cultivars based on the variance in 18 morpho−physiological and biochemical traits grown under control and salt conditions. The first two components explained 54.4% and 20.6% of the variances, respectively. Arrows indicate the strength of the trait influence on the first two PCs. The different color intensities and lengths of the arrows denote the contribution of the traits to the first two components in the PCA. The darker blue and longer arrows indicate a higher contribution, while the darker green and shorter arrows indicate the lower contribution of the variables. PCA−biplot of individual 18 maize genotypes with variables under (**b**) Control condition and under (**c**) Salt condition. The length of the blue arrows indicates the contribution of the attributes to the first two components of PCA. RL (Root Length, cm), SL (Shoot Length, cm), RFW (Root Fresh Weight, g), SFW (Shoot Fresh weight, g), TFW (Total Fresh Weight, g), RDW (Root Dry Weight, g), SDW (Shoot Dry Weight, g), TDW (Total Dry Weight, g), RSR (Root–Shoot Ratio), SPAD (Leaf Greenness, %), A (Photosynthesis rate, µmol CO_2_ m^−2^ s^−1^), RootK (Root K^+^ concentration, mg g^−1^ DW), ShootK (Shoot K^+^ concentration, mg g^−1^ DW), TotalK (Total K^+^ concentration, mg g^−1^ DW), RootNa (Root Na^+^ concentration, mg g^−1^ DW), ShootNa (Shoot Na^+^ concentration, mg g^−1^ DW), TotalNa (Total Na^+^ concentration, mg g^−1^ DW), KNa (K^+^–Na^+^ Ratio).

**Figure 6:**
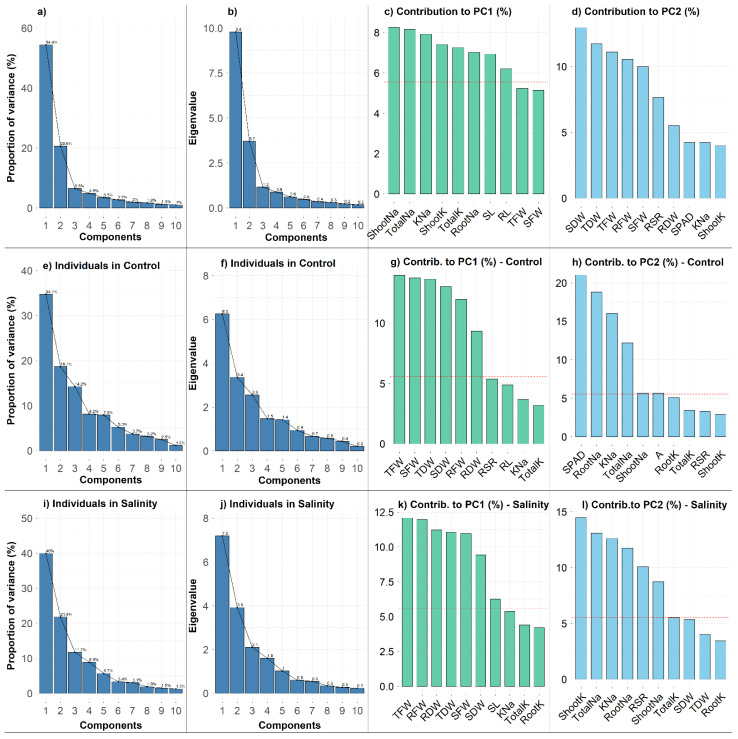
Proportion of variance (%) (**a**,**e**,**i**) and Eigenvalues of first 10 principal components (**b**,**f**,**j**) derived from different PCA-biplots. Contribution (%) of the top 10 measured traits to PC1 (**c**,**g**,**k**) and PC2 (**d**,**h**,**l**). Red dashed lines in the barplots denote reference lines and the variable bars above the reference lines are considered as important in contributing to the dimension. RL (Root Length, cm), SL (Shoot Length, cm), RFW (Root Fresh Weight, g), SFW (Shoot Fresh weight, g), TFW (Total Fresh Weight, g), RDW (Root Dry Weight, g), SDW (Shoot Dry Weight, g), TDW (Total Dry Weight, g), RSR (Root–Shoot Ratio), SPAD (Leaf Greenness), A (Photosynthesis rate, µmol CO_2_ m^−2^ s^−1^), RootK (Root K^+^ concentration, mg g^−1^ DW), ShootK (Shoot K^+^ concentration, mg g^−1^ DW), TotalK (Total K^+^ concentration, mg g^−1^ DW), RootNa (Root Na^+^ concentration, mg g^−1^ DW), ShootNa (Shoot Na^+^ concentration, mg g^−1^ DW), TotalNa (Total Na^+^ concentration, mg g^−1^ DW), KNa (K^+^–Na^+^ Ratio).

The principal component analysis (PCA) was conducted using the experimental dataset including 18 maize cultivars and 18 different variables to reduce the dimensionality of the data and to reveal the potential relationships among the measured traits (Figure 5 and Figure 6). The PCA results showed that the first three principal components (PCs) with eigenvalues >1 accounted for 81.5% of the total variation (Figure 6a,b). Since the first two PCs showed the highest percentage of variance (75.0%), the PCA−biplot was constructed only with the PC1 and PC2 (Figure 5a). The PC1 explained 54.4% of the total variability among traits or individuals and was mostly associated with Shoot Na^+^, Total Na^+^, K^+^–Na^+^ Ratio, Shoot K^+^, Total K^+^, Root Na^+^, SL and RL (Figure 6a,c). The PC2 accounted for an additional 20.6% of the total variability among traits and appeared to be related with SDW, TDW, TFW, RFW, SFW and RSR (Figure 6a,d). The PC3, PC4 and PC5 explained only 6.5%, 4.8% and 3.5% of the phenotypic variations, respectively (Figure 6a). The PC3 was strongly associated with RSR and RDW; PC4 with Root K^+^ and SPAD; and PC5 with *A*. PCA resulted in a clear separation between the control and salt treatments (Figure 5a). The traits grouped in row Cluster-1 (Figure 2) such as Shoot Na^+^, Root Na^+^ and Total Na^+^ represented the major contributor of PC1 and were strongly associated with cultivar Cluster-1. The 10 traits from Cluster-3 (trait cluster) were contributed majorly by the PC1 and PC2, and these traits were also considerably linked to the categorization of the cultivars into salt−tolerant and salt−sensitive groups (Clusters 1–3).

Additionally, PCA−biplots were constructed to illustrate the cultivars’ dispersion in different ordinates based on their differences under control and salt conditions (Figure 5b,c). PC1 and PC2 explained 34.7% and 18.7%, respectively, of the total variability across attributes in the control condition, whereas they explained 40% and 21.8%, respectively, of the total variability in the salt condition (Figure 5b,c and Figure 6e,i). For the first five PCs in control and the first four PCs in salt conditions, the eigenvalues were >1, respectively (Figure 6f,j). In the control condition, PC1 was strongly connected with Na^+^ and K^+^ accumulation traits, but PC2 was strongly associated with biomass-related features (Figure 6g,h). In comparison, biomass traits mostly contributed to PC1, whereas ion accumulation traits had a larger influence on PC2 in stress condition (Figure 6k,l). Overall, the PCA in this experiment indicates that the ion accumulation traits followed by biomass and physiological traits could be used in selection for salt tolerance.

### 3.5. Correlation of Traits

**Figure 7:**
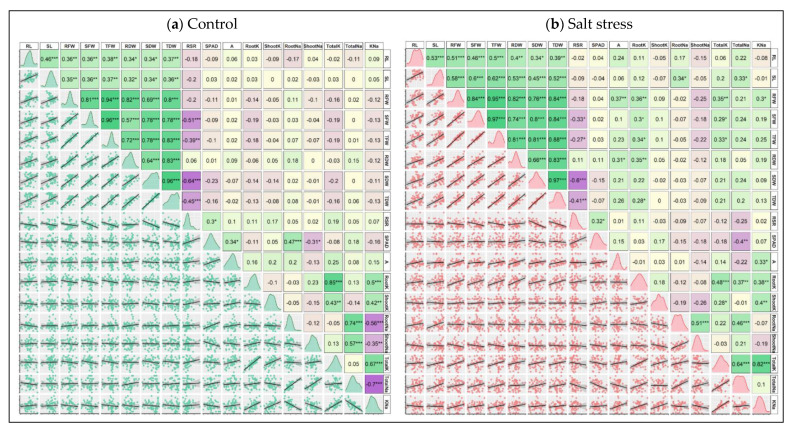
Scatter plot, data distribution and correlation matrix of the 18 examined seedling traits of 18 maize cultivars grown in control (**a**) and salt stress (**b**) conditions. In the upper panels of both figures, green, corn silk and purple boxes denote positive, neutral and negative correlations with correlation coefficient values of 1, 0 and −1, respectively. The increasing color intensities illustrate a higher coefficient. The histograms of data were shown in diagonal panels and the lower panels reflect the scatterplot and trendline of the correlated traits. *, ** and *** indicate 5%, 1% and 0.1% levels of significance, respectively. RL (Root Length, cm), SL (Shoot Length, cm), RFW (Root Fresh Weight, g), SFW (Shoot Fresh weight, g), TFW (Total Fresh Weight, g), RDW (Root Dry Weight, g), SDW (Shoot Dry Weight, g), TDW (Total Dry Weight, g), RSR (Root–Shoot Ratio), SPAD (Leaf Greenness, %), A (Photosynthesis rate, µmol CO_2_ m^−2^ s^−1^), RootK (Root K^+^ concentration, mg g^−1^ DW), ShootK (Shoot K^+^ concentration, mg g^−1^ DW), TotalK (Total K^+^ concentration, mg g^−1^ DW), RootNa (Root Na^+^ concentration, mg g^−1^ DW), ShootNa (Shoot Na^+^ concentration, mg g^−1^ DW), TotalNa (Total Na^+^ concentration, mg g^−1^ DW), KNa (K^+^–Na^+^ Ratio).

To understand the extent of the relationship among the traits, the correlation matrix for control and stress values was made by Pearson correlation analysis (Figure 7a,b). Under control conditions, significant positive correlations among the morphological traits such as RL, SL, RFW, SFW, TFW, RDW, SDW and TDW were observed. The correlation coefficient (*r*) values ranged from 0.32 to 0.96 (Figure 7a). The RSR negatively correlated with the morphological traits and maintained significant correlations with SFW (0.51), TFW (−0.39), SDW (−0.64) and TDW (−0.45). The RSR was positively associated with the other physiological and ion accumulation traits, and the association was significant only with SPAD (0.3) (Figure 7a). The photosynthetic rate (*A*) showed a positive correlation with almost all the traits except SDW, TDW and Shoot Na+. The K^+^–Na^+^ Ratio maintained positive and significant correlations with Root K^+^ (0.5), Shoot K^+^ (0.42) and Total K^+^ (0.67), whereas a significant negative correlation between the K^+^–Na^+^ Ratio and each of Root Na^+^ (−0.56), Shoot Na^+^ (−0.35) and Total Na^+^ (−0.7) was observed (Figure 7a).

Similar to the control condition, the morphological traits under the stress condition showed a significantly positive correlation with *r*-value between 0.34 to 0.97 (Figure 7b). The RSR maintained a negative correlation with almost all traits, and the relationship was significant with SFW, TFW, SDW and TDW (Figure 7b). The *A* positively correlated with all the traits except Root K^+^, Shoot Na^+^ and Total Na^+^. The ion accumulation traits were strongly correlated with each other (Figure 7b). The Shoot Na+ showed negative correlation with all the measured traits except Root Na^+^ (0.51) and Total Na^+^ (0.21) (Figure 7b). Total Na^+^ positively and significantly associated with SL (0.33), Root K^+^ (0.37), Root Na^+^ (0.46) and Total K^+^ (0.64). Apart from this, a significant negative correlation between Total Na^+^ and SPAD (−0.4) was observed (Figure 6b). K^+^–Na^+^ Ratio maintained a strong positive correlation with Total K^+^ (0.82).

### 3.6. Genetic Variability, Heritability and Genetic Advance

The genotypic variance (σ^2^_g_) and phenotypic variance (σ^2^_p_) were ranged from 0.024–39.54 and from 0.03–142.4, respectively (Table 3). The results revealed that the σ^2^_g_ was greater than the interaction variances (genetic vs. salt, σ^2^_gs_) for all traits except SPAD, Root Na^+^, Shoot Na^+^, Total Na^+^ and K^+^–Na^+^ ratio, which suggests that genetic variance had the predominant role in determining these traits (Table 3). Therefore, salt stress had less impact on determining these traits. However, the genetic variance was smaller than the phenotypic variance for all traits. The broad sense heritability (h^2^_bs_) in all measured traits varied from 25.4% for Total Na^+^ to 96.4% for SDW (Table 3). The heritability in the broad sense was categorized as low (0–30%), moderate (30–60%) and high (>60%). Therefore, according to the classification, high h^2^_bs_ was observed in all morphological traits along with *A,* Root K^+^ Shoot K^+^ and Total K^+^, whereas moderate h^2^_bs_ was recorded in SPAD, Root Na^+^, Shoot Na^+^ and K^+^–Na^+^ ratio (Table 3). The magnitude of PCV was found to be slightly higher than the respective GCV for all morphological traits and for Shoot K^+^ (Table 3). Moreover, the narrow magnitude of differences between PCV and GCV were registered in SPAD, *A*, Shoot Na^+^, Root K^+^ and Total K^+^, while the difference was considerable in Root Na^+^, Total Na^+^ and K^+^–Na^+^ ratio (Table 3). The genetic advance (GA) ranged from 0.42 for RDW to 26.28 for TFW. The genetic advance as a percent of mean (GAM) was classified as low (<10%), moderate (10–20%) and high (>20%). Considering this delineation, all the studied traits showed high GAM except SPAD (16.77). The highest GAM was estimated in SDW (85.85) followed by SFW (81.82), RFW (80.59) and TFW (78.88) (Table 3).

## 4. Discussion

Vegetative growth of maize appears to be the most sensitive to salinity [54,55,56], while plants are much less affected at later stages [57]. In the present study, several morpho-physiological and biochemical traits were assessed in 18 hybrid maize cultivars at the early growth stage to evaluate their relative tolerance ability to salt stress. The results of ANOVA (Table S1) revealed highly significant variations among cultivars (C) and between salt treatments (S) for almost all of the examined traits, indicating a genetic difference between the maize cultivars used for salt tolerance.

All morphological and growth traits such as RL, SL, RFW, SFW, TFW, RDW, SDW and TDW were significantly reduced by salt stress in all studied cultivars (Figure 2 and Figure S1). Total fresh and dry masses as the measures of growth maintenance during salt stress were played as driving traits for most of the variations across cultivars. These growth maintenance traits have been widely acknowledged to be a good estimate of salinity tolerance, especially at the early vegetative stage of growth [38].

As 18 cultivars were explored in two different stress treatments, they were subjected to the cluster analysis to visualize the salt resistance group more easily. Hierarchical cluster analysis revealed three distinct clusters for the 18 cultivars studied, and each cluster had six different cultivars (Table 2). Being resistant and moderately resistant, Cluster-1 and Cluster-3 showed lesser and moderate degrees of reduction, respectively, in most growth parameters under salinity as compared to Cluster-2 that showed the highest magnitude of reduction under salinity. Additionally, salt tolerance indices (STI) for the plant morphological and growth parameters showed the magnitude of resistance in the order of Cluster-1 > Cluster-3 > Cluster-2. These results are consistent with many other previous studies [58,59,60,61,62,63,64].

Growth reduction due to salinity occurs at two phases [65]. Immediately after salt application growth reduction occurs due to the osmotic effect, while further growth reduction takes place when excess amounts of salt ions are accumulated in the plant tissues during the second phase of salinity. In this experiment, after two weeks of exposure to salinity, plants showed tip necrosis symptoms at their older leaves. For the maize, it is an indication that plants were already in the second phase of salinity [66].

In this context, plants’ K^+^ and Na^+^ concentrations and their ratios in the root and shoot tissues seem very important indicators to judge salinity resistance. Salt stress boosts excess buildup of rhizospheres Na^+^ and Cl^−^Sodium is the principal toxic ion in maize, and excess Na+ interferes with potassium uptake and transport, leading to disturbance in stomatal regulation and causing water loss and necrosis [67,68]. In the current study, a higher accumulation of sodium and lower accumulation of potassium by all of the cultivars were observed, resulting in a reduced K^+^–Na^+^ ratio under salt conditions (Figure 2). Potassium contents in the roots and shoots of maize decreased due to competition between K^+^ and Na^+^ under salt stress [69,70]. Additionally, necrotic patches form on aged leaves when Na^+^ buildup in guard cells impairs stomatal regulation [71]. Our experiment also showed clear necrosis of the tips of older leaves (Figure S1), which might come from the Na^+^ toxicity. It has been reported that the ability to maintain K^+^ uptake and a high K^+^–Na^+^ ratio under salt stress is a key feature of salt tolerance in plants [72,73]. An increased salinity level substantially raised sodium concentrations in ten maize hybrids and decreased calcium and potassium contents leading to reduced potassium/sodium and calcium/sodium ratios [74]. A study with 19 maize genotypes revealed that salt-tolerant genotypes had appreciably lower sodium accumulation in shoots manifesting higher K^+^–Na^+^ ratio, and suggested that Na^+^ buildup in the shoot is a reliable screening parameter in salt tolerance in the early growth stages of maize [75]. Again, Cluster-1 had relatively better K^+^–Na^+^ ratios under salt stress as compared to the other two clusters, and this may favor the genotypes in Cluster-1 to achieve relatively better growth under salinity.

Additionally, it has been demonstrated that when K^+^ is substituted by Na^+^, chloroplast function is hindered [76]. Ion toxicity in the second phase may directly inhibit photosynthesis and, thus, yield formation [77]. In the present study, leaf greenness (SPAD) and rate of photosynthesis (*A*) were significantly declined due to salt stress. However, the cultivars in Cluster-1 showed less decline in chlorophyll concentrations, although the rate of photosynthesis in both salt-resistant and salt-sensitive clusters was identified to be declined in a similar magnitude (Table 2). The decrease in chlorophyll content under salt stress is a commonly reported phenomenon, and in various studies, chlorophyll concentration has been used as a sensitive indicator of the cellular metabolic state [78]. The degradation of chlorophyll and carotenoid may reduce photosystem (PS) II efficiency and net photosynthetic rate in plants. Several studies have shown a decrease in chlorophyll content under salinity in many plant species due to different reasons, one of which is related to membrane deterioration [79,80]. Carbon fixation is very sensitive to salt stress [81]. Salinity-induced photosynthesis reductions are associated with both stomatal and non-stomatal limitations and their combinations in maize [82]. They also concluded that the reduced gas exchange, as a consequence of limited stomatal conductance and decreased enzyme activities of bundle sheath cells, was responsible for reduced photosynthetic activity in maize plants under salt stress. An increased salt accumulation in older leaves (Figure S1) results in premature declining of leaf greenness, limiting the rate of photosynthesis and, consequently, leading to lower biomass [83].

The relative changes in salt stress, as compared to the control treatments, for all measured traits were expressed as a salt tolerance index (STI) score and used as an indicator for selecting salt-tolerant cultivars. Cluster analysis is practiced by examining large datasets with multiple variables, and this analysis allows grouping of the cultivars with similar traits related to salt tolerance. The 18 maize cultivars showed considerable variations in STI for all measured traits in the present study, and, therefore, the cultivars were grouped into salt–sensitive and salt–tolerant groups by a two-way heatmap clustering pattern using standardized STI values (Figure 3). The cluster analysis separated the tested maize cultivars into three major groups. Cluster-1 consisted of Prabhat, UniGreen NK41, Bisco 51, UniGreen UB100, Bharati 981 and Star Beej 7Star cultivars. The cultivars of this group exhibited the highest degree of salt tolerance, showing higher STI in morphological and physiological traits (blueish) and lower STI in Shoot Na^+^, Root Na^+^ and Total Na^+^ traits (Figure 3). Cluster-2, with six maize cultivars, demonstrated lower STI in almost all traits, and this cluster was categorized as the salt-sensitive cluster. Cluster-3, with the other six cultivars, showed slightly better tolerance than Cluster-2 according to the STI score. The separation and classification of examined traits were also clear. The traits such as K^+^–Na^+^ ratio, dry mass of root and shoot, and Na^+^ and K^+^ contents played a significant role in discriminating salt-tolerant and salt-sensitive groups of maize cultivars (Figure 3). Total K^+^, K^+^–Na^+^ ratio, TFW and TDW were generally higher, whereas Root Na^+^, Shoot Na^+^ and Total Na^+^ were considerably lower in Cluster-1 cultivars than the other two clusters (Figure 4).

PCA was used in this study to identify the most important selection traits for salt tolerance, by using the first and second principal components (Figure 5). PCA–biplot is a type of multivariate analysis that combines traits and objects in two dimensions together and minimizes overlapping variations, facilitating the determination of main characters for selection [42]. The PCA revealed that the traits Shoot Na^+^, Total Na^+^, K^+^–Na^+^ ratio, Shoot K^+^, Total K^+^, Root Na^+^, SDW, TDW, TFW, RFW, SFW, RL and SL contributed more to describing the variation across cultivars (Figure 5 and Figure 6). The increased potassium and decreased sodium contents in roots and shoots resulted in a higher K^+^–Na^+^ ratio. On the other hand, the increased total dry mass was derived from the cumulative increase in RL, SL, RFW, SFW, RDW and SDW. The loading plot for the K^+^–Na^+^ ratio proved unequivocally that it maintained a strong acute angle and, hence, strong positive correlations with the loading plots for K^+^ concentrations in plant parts under salinity conditions.

The correlation pattern in this study also showed a significant and strong positive correlation between TDW and RL, SL, RFW, SFW, TFW, RDW and SDW under both control and salt stress conditions (Figure 7). Similarly, K^+^–Na^+^ ratio maintained a significant positive correlation with Root K^+^, Shoot K^+^ and Total K^+^ and a negative or close to neutral correlation with Root Na^+^, Shoot Na^+^ and Total Na^+^. Interestingly, the root K^+^ level seemed to be a highly dominant parameter that maintained a significantly positive correlation with the majority of biomass-related growth metrics when exposed to salt but not when exposed to control conditions. Additionally, K^+^–Na^+^ ratio formed significantly positive correlations with the rate of photosynthesis but only under salinity (Figure 7). This suggests that increasing the K^+^–Na^+^ ratio may lead to an increase in the rate of photosynthesis of plants under salinity. Physiological traits such as SPAD and *A* exhibited lower contributions in PCA as well as poor correlations with other traits, thereby cutting off from selection criteria at the seedling stage (Figure 5, Figure 6 and Figure 7). Taken together, PCA and correlation analysis results indicate that the morphological and ion accumulation traits, especially TFW, TDW, Total K^+^, Total Na^+^ and K^+^–Na^+^ ratio may be used as evidence in the screening and selection of superior genotypes for salt tolerance. These results are supported by many other previous studies [38,62,72,73,84].

The study of genetic variability and genetic advance provides clear information regarding the extent of variability in a plant population. It gives a relative efficiency measure in genotype selection based on phenotype in a highly variable population [85]. In the present study, the magnitude of genotypic variances was higher than the corresponding interaction variances in almost all of the traits, indicating that the genotypic component of variation was the major contributor to the total variation in the examined traits. The slightly higher PCV than GCV in morphological traits and Shoot K^+^ reflect the presence of an environmental influence, to some extent, in the phenotypic expression of the characters (Table 3). Thus, the phenotypic performance of these characters would be effective for selection in consideration of genetic improvement [86]. The broad-sense heritability (h^2^_bs_) estimates the relative magnitude of genetic or environmental variation in a population, and a high h^2^_bs_ indicates less environmental influence in the observed variations. The high h^2^_bs_ (>60%) was found in all morphological traits, A, Root K^+^, Shoot K^+^ and Total K^+^, indicating that considerable genetic variation is present in these traits (Table 3). However, heritability alone is not enough to determine the selection, and a combination of h^2^_bs_, GCV and genetic advance (GA) is, therefore, most useful for effective and reliable selection providing additive gene action. Johnson et al. [52] suggested the simultaneous consideration of heritability and GA estimation because high heritability may not always be associated with high GA. These traits may respond to phenotypic selection and could be improved through heterosis breeding [87,88]. The traits with a combination of high h^2^_bs_, GCV and GAM (>20%) in this study indicate that the variation in these examined traits is largely due to the genetic factors, and these traits can be used as reliable screening criteria for the evaluation of salt-tolerant maize genotypes.

## 5. Conclusions

Establishing salt–tolerant cultivars is the key approach to mitigating soil salinity under global climate change. A comprehensive understanding of the genotypes–traits relationship is needed for selection. A total of 18 hybrid maize cultivars were screened based on diverse morpho–physiological and biochemical traits in this study. The results revealed a considerable genotypic variability in response to salt stress in the maize seedlings. The cultivars Prabhat, UniGreen NK41, Bisco 51, UniGreen UB100, Bharati 981 and Star Beej 7Star exhibited greater salt tolerance characterizing higher plant biomass, lower Na^+^ and higher K^+^ accumulation, and maintaining an increased K^+^–Na^+^ ratio. Integration of high heritability (h^2^_bs_, >60%) and genetic advance (GAM, >20%) was recorded in 13 measured traits indicating the variations among these traits were largely due to genetic factors. The highlighted salt-tolerant maize cultivars could be used for cultivation in the coastal saline soils with further field trials. Some traits such as Total Na^+^, Total K^+^ contents, K^+^–Na^+^ Ratio, TFW, SDW and TDW could be effectively used for the selection in salt-tolerant maize cultivar development at the early seedling stage. The study could uphold the understanding of the methods to utilize the existing plant genetic resources for the improvement of salt-tolerant maize cultivars.

## Figures and Tables

**Table 1:** List of 18 hybrid maize cultivars used in the study for screening of salt tolerance.

Sl.	Cultivar’s Name	Sl.	Cultivar’s Name
1.	Bharati 40	10.	Essence Platinum
2.	Bharati 702	11.	Getco 901
3.	Bharati 888	12.	Kaveri Jambo
4.	Bharati 981	13.	Kaveri 244
5.	Bisco 51	14.	Prabhat
6.	Bisco Prince	15.	Star Beej 7Star
7.	Dekalb Elite	16.	Star Beej Kohinoor
8.	Dekalb Super 900M	17.	Unigreen NK41
9.	Dekalb 900M Gold	18.	Unigreen UB100

**Table 2:** Cluster (row) means of measured traits in 18 maize cultivars grown under control and salt conditions.

Traits	Cluster-1		Cluster-2		Cluster-3	
	Control	Salt	Control	Salt	Control	Salt
Root Length (cm)	46.8 ± 1.3 a	37.5 ± 0.9 b	46.6 ± 2.4 a	32.1 ± 1.3 b	47.7 ± 1.3 a	35.1 ± 1.3 b
Shoot Length (cm)	55.9 ± 1.2 a	44.8 ± 0.8 b	51.4 ± 1.5 a	37.9 ± 1.8 c	55.1 ± 0.7 a	41.3 ± 1.4 bc
Root Fresh Weight (g)	19.2 ± 0.7 a	16.1 ± 0.7 ab	15.7 ± 2.1 ab	11.7 ± 1.5 b	16.7 ± 1.5 ab	11.3 ± 0.9 b
Shoot Fresh Weight (g)	22.2 ± 0.8 a	18.7 ± 0.7 ab	17.0 ± 2.4 abc	10.9 ± 1.5 c	22.8 ± 2.0 a	13.3 ± 0.9 bc
Total Fresh Weight (g)	41.3 ± 1.2 a	34.9 ± 1.2 ab	32.7 ± 4.4 abc	22.6 ± 2.9 c	39.6 ± 3.5 a	24.6 ± 2.1 bc
Root Dry Weight (g)	0.7 ± 0.03 a	0.6 ± 0.03 ab	0.7 ± 0.08 a	0.4 ± 0.05 b	0.6 ± 0.05 ab	0.4 ± 0.04 b
Shoot Dry Weight (g)	1.4 ± 0.1 a	1.2 ± 0.09 ab	1.2 ± 0.14 ab	0.8 ± 0.1 b	1.2 ± 0.1 ab	0.8 ± 0.07 b
Total Dry Weight (g)	2.2 ± 0.11 a	1.8 ± 0.09 ab	1.9 ± 0.21 a	1.3 ± 0.14 b	1.8 ± 0.13 ab	1.2 ± 0.11 b
Root–Shoot Ratio	0.6 ± 0.05 ab	0.6 ± 0.06 ab	0.7 ± 0.06 a	0.6 ± 0.04 ab	0.5 ± 0.03 b	0.6 ± 0.04 ab
Leaf Greenness (SPAD)	32.0 ± 0.8 b	28.4 ± 0.7 c	35.9 ± 0.9 a	27.3 ± 0.8 c	32.9 ± 0.8 ab	27.2 ± 0.7 c
Photosynthetic Rate (*A*, µmol CO_2_ m^−2^s^−1^)	7.0 ± 0.3 ab	5.3 ± 0.1 c	7.9 ± 0.2 a	6.0 ± 0.5 bc	7.3 ± 0.3 a	5.2 ± 0.3 c
Root K^+^ (mg g^−1^ DW)	16.3 ± 1.9 ab	14.4 ± 2.0 bc	17.5 ± 1.5 ab	9.4 ± 1.7 d	18.4 ± 2.8 a	12.1 ± 2.0 cd
Shoot K^+^ (mg g^−1^ DW)	21.3 ± 1.7 a	12.5 ± 0.9 b	20.8 ± 1.08 a	11.7 ± 1.1 b	20.7 ± 1.2 a	11.6 ± 1.0 b
Total K^+^ (mg g^−1^ DW)	37 ± 2.3 a	27.0 ± 3.2 b	38.3 ± 1.7 a	21.0 ± 2.2 c	39.1 ± 2.9 a	23.7 ± 2.0 bc
Root Na^+^ (mg g^−1^ DW)	0.84 ± 0.13 c	2.86 ± 0.26 b	1.15 ± 0.21 c	3.89 ± 0.46 a	0.88 ± 0.12 c	4.02 ± 0.57 a
Shoot Na^+^ (mg g^−1^ DW)	2.23 ± 0.11 c	7.67 ± 0.20 b	2.21 ± 0.14 c	8.14 ± 0.21 a	2.27 ± 0.17 c	8.5 ± 0.37 a
Total Na^+^ (mg g^−1^ DW)	3.1 ± 0.20 c	10.52 ± 0.37 b	3.37 ± 0.22 c	12.03 ± 0.48 a	3.14 ± 0.18 c	12.5 ± 81 a
K^+^–Na^+^ Ratio	12.4 ± 1.06 a	2.6 ± 0.3 b	11.5 ± 1.0 a	1.8 ± 0.21 b	12.5 ± 1.1 a	1.9 ± 0.12 b

Values are mean ± SEM. Row means with different letters are significantly different at 5% levels of probability.

**Table 3:** Estimates of variance components, heritability, genotypic and phenotypic coefficients of variance, genetic advance and genetic advance as percent of the mean for 18 maize cultivars grown under two salinity levels.

Traits	Grand Mean	σ^2^_g_	σ^2^_gs_	σ^2^_p_	h^2^_bs_	GCV	PCV	GA	GAM
Root Length (cm)	41.0	25.23	9.03	31.07	81.2	12.26	13.60	14.75	36.00
Shoot Length (cm)	47.7	25.05	4.77	28.47	88.0	10.49	11.18	15.79	33.09
Root Fresh Weight (g)	15.1	29.84	1.65	31.43	94.9	36.07	37.02	12.20	80.59
Shoot Fresh Weight (g)	17.5	39.54	8.57	44.84	88.2	35.95	38.28	14.31	81.82
Total Fresh Weight (g)	32.6	131.2	17.48	142.4	92.2	35.10	36.56	25.74	78.88
Root Dry Weight (g)	0.6	0.03	0.01	0.04	87.0	29.96	32.11	0.42	71.25
Shoot Dry Weight (g)	1.1	0.19	0.01	0.19	96.4	39.07	39.80	0.95	85.85
Total Dry Weight (g)	1.7	0.34	0.02	0.35	95.8	34.30	35.04	1.31	77.17
Root–Shoot Ratio	0.6	0.024	0.0004	0.03	88.3	26.28	27.96	0.37	63.77
Leaf Greenness (SPAD)	30.6	3.2	3.387	6.00	53.3	5.84	8.00	5.13	16.77
Photosynthetic Rate (*A*, µmol CO_2_ m^−2^ s^−1^)	6.5	0.48	0.345	0.80	60.2	10.72	13.83	1.98	30.66
Root K^+^ (mg g^−1^ DW)	20.9	5.38	4.41	8.39	64.1	15.79	19.73	6.13	41.74
Shoot K^+^ (mg g^−1^ DW)	5.8	0.39	−1.25	0.52	73.9	3.78	4.40	7.49	45.53
Root Na^+^ (mg g^−1^ DW)	8.3	0.08	0.31	0.25	32.1	12.56	22.14	0.99	43.41
Shoot Na^+^ (mg g^−1^ DW)	2.9	0.03	0.05	0.08	41.3	3.44	5.36	2.53	48.95
Total K^+^ (mg g^−1^ DW)	10.5	6.00	3.40	9.27	64.7	7.87	9.78	11.23	36.08
Total Na^+^ (mg g^−1^ DW)	2.4	0.12	0.60	0.45	25.4	4.56	9.05	2.28	30.69
K^+^–Na^+^ Ratio	4.9	0.21	0.25	0.57	37.0	6.44	10.60	3.98	56.01

σ^2^_g_, σ^2^_gs_ and σ^2^_p_ denote Genetic, Genetic × Salinity and Phenotypic variances, respectively; GCV and PCV = Genotypic and Phenotypic coefficient of variability and h^2^_bs_ = Heritability in broad sense; GA = Genetic Advance; GAM = Genetic Advance as Percent of Mean.

## Data Availability

The data that support the findings of this study are available from the corresponding authors upon reasonable request.

## Supplementary Materials

The following are available online at https://www.mdpi.com/article/10.3390/plants10112549/s1, Table S1: Mean square values of measured traits obtained from two-way ANOVA (analysis of variance) in eighteen hybrid maize cultivars grown under control and salt stress environments. Figure S1: Seedlings of some salt-tolerant (1–4) and salt-sensitive (5–12) maize hybrid cultivars grown under 0 (left) and 12 dS m^−1^ (right) growth conditions. (Figure legends: 1-UniGreen NK41, 2-Bisco 51, 3-Star Beej 7Star, 4-Prabhat, 5-Kaveri 244, 6- Dekalb Elite, 7-Dekalb 900M Gold, 8-Kaveri Jambo, 9-Dekalb Super 900M, 10-Essence Platinum, 11-Getco 901, 12-Bharati 888).
