# Supplementary material for: Characterization of Maize Hybrids (Zea mays L.) for Detecting Salt Tolerance Based on Morpho-Physiological Characteristics, Ion Accumulation and Genetic Variability at Early Vegetative Stage
Source: Plants (Basel). 2021 Nov 22;10(11):2549. doi: 10.3390/plants10112549 (PMC8623748; doi:10.3390/plants10112549)
Supplement: Supplementary file 1 [file plants-10-02549-s001.zip › plants-1437670-supplementary.pdf]

Table S1: Mean square values of measured traits obtained from two-way ANOVA (analysis of variance) in eighteen hybrid maize cultivars grown under control and salt stress environments.

Traits	Symbol	Cultivar (C)	Salt Stress (S)	Interaction (C×S)
Root Length (cm)	RL	186.4^{***}	3978.5^{***}	35^{***}
Shoot Length (cm)	SL	170.8^{***}	4421.1^{***}	20.5^{***}
Root Fresh Weight (g)	RFW	188.59^{***}	465.76^{***}	9.55^{*}
Shoot Fresh Weight (g)	SFW	269.06^{***}	1085.70^{***}	31.85^{***}
Total Fresh Weight (g)	TFW	854.14^{***}	2973.67^{***}	66.85^{***}
Root Dry Weight (g)	RDW	0.216^{***}	1.03^{***}	0.028^{**}
Shoot Dry Weight (g)	SDW	1.16^{***}	2.75^{***}	0.0423^{**}
Total Dry Weight (g)	TDW	2.1184^{***}	7.138^{***}	0.0887^{**}
Root Shoot Ratio	RSR	0.1605^{***}	0.0152^{NS}	0.0188^{NS}
Leaf Greenness (%)	SPAD	35.99^{***}	960.63^{***}	16.8^{**}
Photosynthetic Rate (μmol CO_2 m^{-2} s^{-1})	*A*	4.775^{***}	96.83^{***}	1.902^{*}
Root K^+ (mg g^{-1} DW)	Root K^+	50.3^{***}	793.3^{***}	18.08^{***}
Shoot K^+ (mg g^{-1} DW)	Shoot K^+	3.14^{NS}	2196^{***}	0.82^{NS}
Root Na^+ (mg g^{-1} DW)	Total K^+	55.6^{***}	5628^{***}	19.6^{*}
Shoot Na^+ (mg g^{-1} DW)	Root Na^+	1.52^{***}	187.7^{***}	1.032^{***}
Total K^+ (mg g^{-1} DW)	Shoot Na^+	0.46^{***}	925.5^{***}	0.27^{**}
Total Na^+ (mg g^{-1} DW)	Total Na^+	2.72^{***}	1947^{***}	2.03^{***}
K^+–Na^+ Ratio	K^+–Na^+ ratio	3.41^{**}	2734^{***}	2.15^{NS}

*, ** and *** denote significant at 5%, 1% and 0.1% levels of probability, respectively. NS = Non–Significant

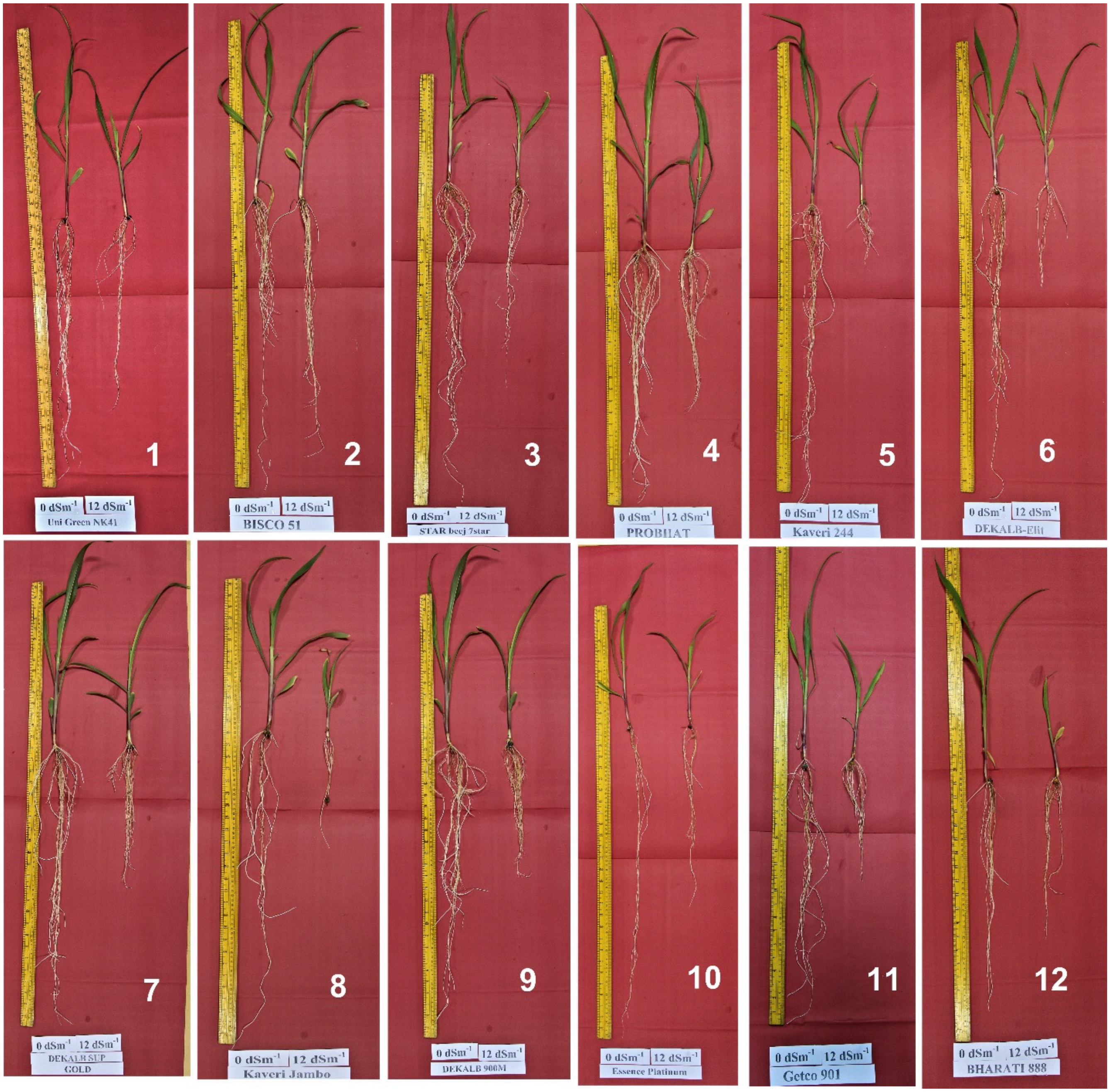

Figure S1: Seedlings of some salt–tolerant (1–4) and salt–sensitive (5–12) maize hybrid cultivars grown under 0 (left) and 12 dS m^{-1} (right) growth conditions. *(Figure legends: 1- UniGreen NK41, 2-Bisco 51, 3-Star Beej 7Star, 4-Prabhat, 5-Kaveri 244, 6- Dekalb Elite, 7-Dekalb 900M Gold, 8-Kaveri Jambo, 9-Dekalb Super 900M, 10-Essence Platinum, 11-Getco 901, 12-Bharati 888)*
